# Red Fox (*Vulpes vulpes*) and Wolf (*Canis lupus*) as a Reservoir of *Cryptosporidium* spp. and *Giardia intestinalis* in Poland

**DOI:** 10.3390/pathogens14050500

**Published:** 2025-05-20

**Authors:** Dorota Dwużnik-Szarek, Ewa Julia Mierzejewska, Korneliusz Kurek, Małgorzata Krokowska-Paluszak, Patrycja Opalińska, Łukasz Stańczak, Grzegorz Górecki, Anna Bajer

**Affiliations:** 1 Department of Eco-Epidemiology of Parasitic Diseases, Institute of Developmental Biology and Biomedical Sciences, Faculty of Biology, University of Warsaw, Miecznikowa 1, 02-096 Warsaw, Poland; ewajuliamierzejewska@gmail.com (E.J.M.); a.bajer2@uw.edu.pl (A.B.); 2 The Masurian Center for Biodiversity and Nature Education in Urwitałt, University of Warsaw, Urwitałt 1, 11-730 Mikołajki, Poland; k.kurek@uw.edu.pl; 3 Faculty of Agricultural and Forestry, University of Warmia and Mazury in Olsztyn, 10-719 Olsztyn, Poland; malgorzata.krokowska.paluszak@uwm.edu.pl; 4 Department of Game Management and Forest Protection, University of Life Sciences, 60-625 Poznań, Poland; opalinska.p16@gmail.com (P.O.); stanczak.luk@gmail.com (Ł.S.); grzegorz.gorecki@up.poznan.pl (G.G.)

**Keywords:** intestinal protozoan parasites, wild canids, natural area, Apicomplexa, Flagellate, zoonotic reservoir

## Abstract

Infections with zoonotic pathogens have received increasing attention in recent years, as reflected in the literature of both veterinary and human medicine. *Cryptosporidium* and *Giardia* are recognised as the principal causes of waterborne outbreaks worldwide, but there is still limited data on the role of wild carnivores, such as red foxes and wolves, as reservoir hosts and in disseminating these pathogens in the environment. The aim of the current project was to analyse the prevalence and abundance of *Cryptosporidium* and *Giardia* infections in foxes from seven voivodeships and in wolves from the Warmia-Masuria Voivodeship in Poland and to conduct a phylogenetic analysis of the detected parasites. For the detection of both parasites, we used the commercial immunofluorescent assay MeriFluor *Cryptosporidium*/*Giardia*. For *Cryptosporidium* detection we also applied modified Ziehl–Neelsen (ZN) staining of faecal smears and, following PCR amplification, sequenced the *18S* rDNA locus. For *Giardia* detection, we sequenced the glutamate dehydrogenase (*gdh*) gene. In total, 117 and 69 faecal samples obtained from red foxes and wolves, respectively, were screened for the presence of *Cryptoporidium*/*Giardia*. In red foxes, prevalence was 38.5% and 15.4% for *Cryptosporidium* spp. and *G. intestinalis*, respectively. In wolves, the prevalence of *Cryptosporidium* spp. was 14.5%, and only one sample was *Giardia*-positive. *Cryptosporidium canis*, *Cryptosporidium* sp. vole genotype, *C. baileyi* and *Cryptosporidium* sp. were identified in red foxes, while *C. canis* and *Cryptosporidium* sp. were detected in wolves. Our results indicate that red foxes and grey wolves act as reservoir hosts of *Cryptosporidium* spp. and *G. intestinalis* in natural areas in Poland.

## 1. Introduction

Wild animals can act as hosts for parasites of medical and veterinary significance and thereby constitute a direct or indirect source of infection for humans and domestic/farmed animals [1,2,3]. In Europe, the red fox (*Vulpes vulpes*) is recognised as an important host for a range of parasite species, including ectoparasites, helminths and bacteria [4,5,6,7]. Foxes’ adaptability to local environmental characteristics and their vagility facilitate the dispersal of parasitic organisms that they carry to new non-endemic areas for the organisms concerned [8,9]. In Poland, foxes are regarded legally as game animals, with a population size estimated at 200,000 individuals in 2023 [10].

The other wild canid occurring in Poland is the grey wolf (*Canis lupus*). The current legal status of wolves is regulated by the Act of 16 April 2004, on Nature Protection (consolidated text, Journal of Laws of 2013, item 627, as amended) and the Regulation of 16 December 2016 (Ministry of the Environment), on protection of animal species (Journal of Laws 2016, item 2183). According to these regulations, and in contrast to red foxes, grey wolves are under strict protection. The population of this predator was lower than that of red foxes in Poland in 2019 and was estimated at about 2000 individuals (data from the Chief Inspectorate of Environmental Protection, with responsibility for monitoring protected species in Poland). Wolves live mainly in forests [11,12] and mostly avoid contact with humans. In Poland, red deer (*Cervus elaphus*), roe deer (*Capreolus capreolus*), and wild boar (*Sus scrofa*) constitute the main prey of wolves [13,14,15]. In northwestern Poland the mean pack size varies between 3.5 and 5.6 individuals, but packs comprising up to 8 individuals have also been recorded [16].

The range of parasites of wolves in Europe is still poorly documented, although recent studies have confirmed the presence of *Toxoplasma gondii* [17], *H. canis* [18] and *Babesia canis* [19] in these populations. Much less is known about the intestinal protozoa, such as *Cryptosporidium* and *Giardia* spp., carried by wild canid species. Both *Cryptosporidium* spp. and *G. intestinalis* have been recorded in faecal samples from foxes in Italy, Norway, Spain and Slovakia [20,21,22,23]. However, there is still only very limited epidemiological data on the role of red foxes as reservoirs of both protozoa species in Poland [24], and only a few cases of infection have been documented in wolves [25,26].

Both parasites are a common cause of diarrhoea in immunocompromised humans, especially HIV-positive and AIDS patients, transplant patients, children with primary immunodeficiencies and the elderly [27,28,29,30]. *Cryptosporidium* and *Giardia* infections have been observed also in patients suffering from gastrointestinal cancers [27,30,31]. Waterborne outbreaks of cryptosporidiosis and giardiasis are commonly reported worldwide, principally but not exclusively, in developing countries or countries with low hygiene standards [32,33,34,35,36]. In humans, *C. parvum* and *C. hominis* are the most common species causing severe cryptosporidiosis [29,37,38,39], but with the introduction of highly sensitive and specific molecular diagnostic tools in recent decades, increasing cases of cryptosporidiosis attributable to zoonotic species, such as *C. canis*, *C. felis* and *C. meleagridis*, are now being observed [28,40,41]. *Giardia intestinalis* is known to comprise a range of genotypes, referred to as assemblages, and of these assemblages A and B constitute the main agents of giardiasis in human populations [42,43].

The aims of the presence study were (i) determination of the prevalence and abundance of *Cryptosporidium*/*Giardia* infections in red foxes among seven voivodeships; (ii) determination of the prevalence of both pathogens in grey wolf populations; (iii) comparison of the prevalence of *Cryptosporidium*/*Giardia* infection between the two canid species; (iv) genetic identification of detected pathogens; (v) comparison of abundance between *Cryptosporidium* species in red fox population.

## 2. Materials and Methods

### 2.1. Collection of Faecal Samples from Foxes

**Figure 1:**
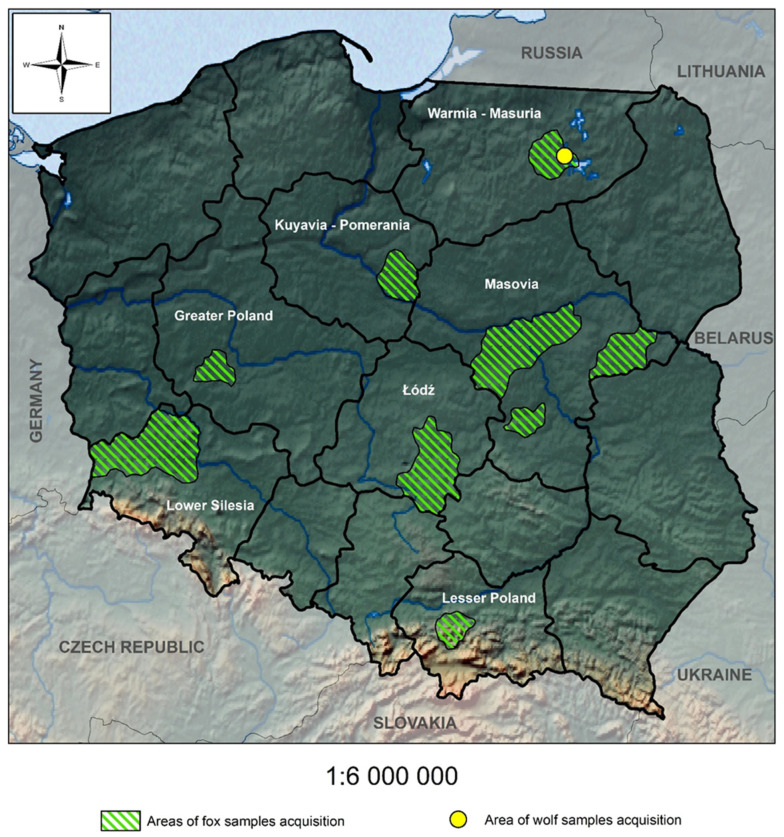
Regions where samples (red fox and wolf) were collected.

Red foxes (carcasses) were obtained during the autumn/winter hunting seasons of 2015/2016, 2016/2017 and 2017/2018 from seven voivodeships: Lower Silesia (17 specimens), Kuyavia-Pomeranian (17), Łódź (28), Masovia (20), Lesser Poland (8), Warmia-Masuria (20), and Greater Poland (7) (Figure 1) [5]. Autopsies of gastrointestinal tracts were conducted in the Department of Eco-Epidemiology of Parasitic Diseases at the Faculty of Biology, University of Warsaw. During autopsy, faecal samples were collected directly from the rearmost parts of the intestines (colon or rectum) into 50 mL Falcon test tubes and frozen at a temperature of −20 °C for further examinations.

### 2.2. Collection of Faecal Samples from Wolves

Wolf faecal samples were collected from the environment in the Warmia-Masuria Voivodeship close to the Urwitałt UW field station (53.80932 N, 21.64764 E) during monitoring of wolf activities and diet studies between 2021 and 2023 (Figure 1). Samples were collected from fresh (up to a few days old) scats into Ziplock bags and frozen at −20 °C for further examinations [44]. Faeces were identified based on morphological features (size, structure, smell). Questionable samples were genetically verified or rejected.

### 2.3. The Modified Ziehl–Neelsen Staining of Faecal Smears

Faecal smears from stool specimens were air-dried, fixed in methanol, and stained by the Ziehl–Neelsen staining protocol for *Cryptosporidium* spp. detection [45]. Stained smears were immersed in DPX, covered with a coverslip and viewed under a light microscope at the 400× magnification. Oocysts were identified based on their size, tint and general morphology.

### 2.4. Immunofluorescence Assay (IFA) MeriFluor Cryptosporidium/Giardia

Faecal samples ranging from 0.3 to 1.0 g were thoroughly homogenised in small amounts of phosphate buffered saline (PBS) pH = 7.2. The suspension was centrifuged at 1900× *g* for 10 min. The resulting sediment was washed and condensed by the modified sucrose flotation method [46]. From the final solution, 10 µL was used for IFA. The indirect immunofluorescence assay Merifluor *Cryptosporidium*/*Giardia* (Meridian Diagnostics, Cincinnati, OH, USA) was implemented according to the manufacturer’s recommendations. IFA slides were examined under an Olympus BX50F immunofluorescence microscope (Olympus Optical Co., Tokyo, Japan) (400× magnification at 490–500 nm wavelength). Identification was made by comparison with positive control samples provided in the kit. Abundance of infection was estimated by the total number of (oo)cysts detected per well, multiplied by 400 (dilution factor × 100 = (oo)cysts per mL) to give the number per mL of concentrated sediment [46].

### 2.5. Molecular Methods

DNA was extracted from faecal samples using the QIAamp PowerFaecal ProDNA Kit (Qiagen, Hilden, Germany) according to the manufacturer’s protocol and then stored at −20 °C until molecular analysis. For the detection of *Cryptosporidium* spp., nested PCR amplification of a 600 bp fragment of *18S* rDNA was conducted, using previously described primers [47]. The primers used for the first reaction were SHP1 5′-ACC TAT CAG CTT TAG ACG GTA GGG TAT-3′ and SHP2 5′-TTC TCA TAA GGT GCT GAA GGA GTA AGG-3′. For the second amplification, primers SHP3 5′-ACA GGG AGG TAG TGA CAA GAA ATA ACA-3′ and SSU-R3 5′-AAG GAG TAA GGA ACA ACC TCC A-3′ were used. Initial and nested PCR conditions were modified as follows: initial denaturation at 94 °C for 3 min, 35 cycles of denaturation at a temperature of 94 °C for 45 s, primer annealing at 58 °C for 45 s and elongation at 72 °C for 60 s, with final elongation at 72 °C for 7 min.

For *G. intestinalis* detection, a semi-nested PCR was performed using three primers: external forward primer: 5′ TCA ACG TYA AYC GYG GYT TCC GT-3′, internal forward primer: 5′ CAG TAC AAC TCY GCT CTC GG 3′ and reverse primer: 5′ GTT RTC CTT GCA CAT CTC C-3′ to amplify a 432 bp fragment of the glutamate dehydrogenase (*gdh*) gene [48]. For the first reaction, the conditions were as follows: initial denaturation at 94 °C for 5 min, 30 cycles of denaturation at a temperature of 94 °C for 30 s, primer annealing at 61 °C for 20 s, elongation at 68 °C for 20 s, with final elongation at 68 °C for 7 min. Nested PCR conditions were initial denaturation at 94 °C for 5 min, 15 cycles of denaturation at 94 °C for 30 s, primer annealing at 60 °C for 20 s, elongation at 65 °C for 20 s, with final elongation at 65 °C for 7 min. In the second step, 1 µL of the PCR product obtained in the first PCR was used as a template [48].

The DNA of *G. intestinalis* and *C. muris* were used as positive controls. Negative controls were performed in the absence of template DNA.

PCR products were subjected to electrophoresis on a 1.5% agarose gel, stained with Midori Green stain (Nippon Genetics, GmbH, Düren, Germany) and were sequenced by a private company (Genomed S.A., Gdańsk, Poland). Sequence alignments and analyses were carried out using BLAST-NCBI 2.10.0+ (the National Center for Biotechnology Information—Basic Local Alignment Sequence Tool) and MEGA 11 software.

Total prevalence of *Cryptosporidium* was calculated based on the combined results of three applied methods (IFA, Z-N, nested PCR). A sample was considered positive if at least one method provided a positive result. Samples were considered positive for *Giardia* if at least one of two methods (IFA or PCR) yielded a positive result. Because of limited material quantity, samples obtained from wolves were processed only by molecular protocols.

### 2.6. Phylogenetic Analysis

All PCR products (*Giardia* and *Cryptosporidium*) were commercially sequenced by the Genomed company (Warsaw, Poland). Sequence alignments and analyses were carried out using BLAST-NCBI 2.10.0+ and MEGA 11 software [49]. Phylogenetic analyses were performed using the Maximum Likelihood method of tree construction. The evolutionary model was chosen in accordance with the data (following the implemented model test in MEGA 11) and bootstrapped over 1000 randomly generated sample trees. Representative *18S* rDNA sequences of *Cryptosporidium* obtained in this study were deposited in GenBank under accession numbers: PV545563, PV546873, PV550074, PV554187, PV565730, PV566746, and PV566908.

### 2.7. Statistical Analysis

For the analysis of prevalence (% infected), we applied maximum likelihood techniques based on log-linear analysis of contingency tables in the IBM SPSS v. 21 software package (IBM Corporation; institutional licence purchased by the University of Warsaw, Warsaw, Poland). HOST SPECIES (two levels: fox and wolf), VOIVODESHIP of host (fox) origin (seven levels: Masovia, Lódź, Kuyavia-Pomerania, Greater Poland, Lower Silesia, Lesser Poland, Warmia-Masuria), SEX of foxes (males and females), and DETECTION METHOD (three levels: IFA, Z-N, PCR) were used as the factors in models with the presence (1) or absence (0) of (oo)cysts of *Cryptosporidium*/*Giardia* considered as a binary factor and referred to as INFECTION. For each level of analysis in turn, beginning with the most complex model, involving all possible main effects and interactions, those combinations that did not contribute significantly to explaining variation in the data were eliminated in a stepwise fashion beginning with the highest level interaction (backward selection procedure). A minimum sufficient model was then obtained, for which the likelihood ratio of χ^2^ was not significant, indicating that the model was sufficient in explaining the data. The importance of each term in interactions involving INFECTION in the final model was assessed by the probability that its exclusion would alter the model significantly, and these values are given in the text, assessed by a likelihood ratio test between nested models with and without each factor of interest. General linear models (GLMs in SPSS v.21) were used for the analysis of mean abundance of oocysts/cysts, using models with normal errors, incorporating HOST SPECIES, VOIVODESHIP of HOST origin and SEX of foxes as fixed factors. Means are presented with the standard error of the mean (S.E.) [5].

## 3. Results

### 3.1. Foxes

Focal samples from 117 foxes in total (65 males and 52 females) were examined by all three methods for *Cryptosporidium* and by two methods for *Giardia* detection. *Cryptosporidium* spp. and *G. intestinalis* were found in 38.5% and 15.4% of foxes, respectively. The highest number of *Cryptosporidium*-positive samples was detected by the modified Ziehl–Neelsen staining of faecal smears (34 samples), then by the immunofluorescence assay (IFA) (26 samples) and molecular methods (10 samples) (detection method × *Cryptosporidium* INFECTION: *χ*^2^_2_ = 5.26, *p* = 0.022). Three *Cryptosporidium*-positive samples were confirmed by three methods (Z-N+IFA+PCR), 19 by two methods (15 by Z-N+IFA; one by IFA+PCR; three by Z-N+PCR); and 23 by one method (13 by Z-N; 7 by IFA; three by PCR). *Giardia intestinalis* was detected by immunofluorescence assay (IFA; 17 samples) and molecular methods (1 sample).

#### 3.1.1. *Cryptosporidium* spp.

**Figure 2:**
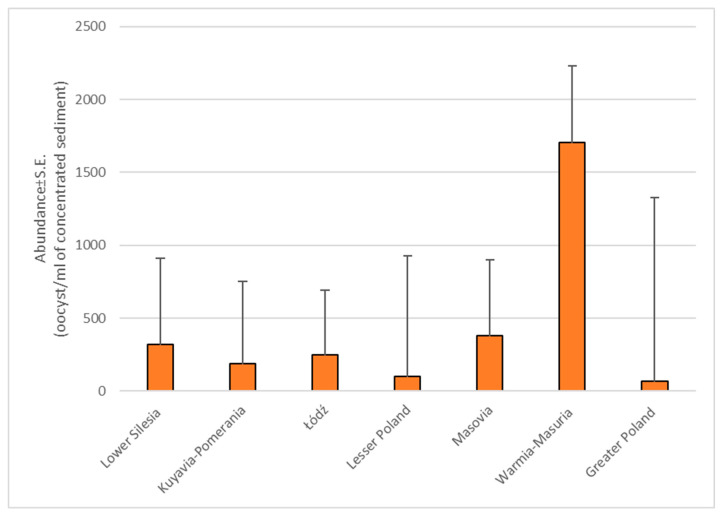
Mean abundance of *Cryptosporidium* spp. between voivodeships.

Similar prevalence of *Cryptosporidium* spp. was observed in male and female foxes: 35.4% vs. 42.3%, respectively (NS). There were some differences in *Cryptosporidium* spp. prevalence between animals obtained from different voivodeships (VOIVODESHIP × *Cryptosporidium* INFECTION: *χ*^2^_6_ = 10.03, *p* = 0.12). The highest prevalence was observed in foxes from the Masovia Voivodeship, and the lowest in Lower Silesia (Table 1). Overall abundance of *Cryptosporidium* spp. infection was 428.84 ± 274.53 oocysts/1 mL of concentrated sediment, and this was similar for males (628 ± 372.31) and females (285 ± 120.00; NS). No statistically significant differences were observed in the abundance of *Cryptosporidium* spp. infection between foxes from the different voivodeships (main effect of voivodeship on *Cryptosporidium* spp. abundance of infection (NS)). Nevertheless, the highest mean abundance of *Cryptosporidium* spp. infection was found in animals from the Warmia-Masuria Voivodeship (1600 ± 516.54), while the abundance of *Cryptosporidium* spp. was lower and similar in foxes from the remaining six voivodeships (Figure 2).

#### 3.1.2. *Giardia intestinalis*

The prevalence of *G. intestinalis* was identical in male and female foxes (15.4%; NS). The percentage of infected animals was also identical in the Masovia and Warmia-Masuria Voivodeships (Table 1). *Giardia intestinalis* infection was detected in foxes from Lower Silesia, Łódź and the Kuyavia-Pomerania Voivodeships. None of the foxes from the Lesser Poland and Greater Poland Voivodeships were positive for *G. intestinalis* (VOIVODESHIP × *Giardia* INFECTION: *χ*^2^_6_ = 13.73, *p* = 0.03).

**Figure 3:**
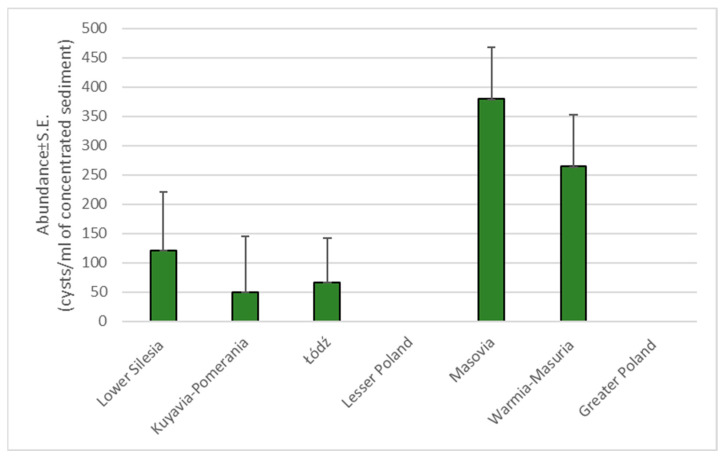
Mean abundance of *G. intestinalis* spp. between voivodeships.

Overall mean abundance of *G. intestinalis* infection was 126.08 ± 46.25 cysts/1 mL of concentrated sediment, and this was similar in males (128.6 ± 53.10) and females (123.55 ± 75.75; NS). Abundance of *G. intestinalis* differed between voivodeships: the highest abundance was observed in animals from the Masovia and Warmia-Masuria Voivodeships, and the lowest for those from the Kuyavia-Pomerania Voivodeship (main effect of voivodeship on *Giardia* abundance: *F*_6,116_ = 2.09, *p* = 0.006; Figure 3).

### 3.2. Wolves

In total, 69 wolf faecal samples from the Warmia-Masuria Voivodeship were screened for *Cryptosporidium*/*Giardia* by molecular methods. The prevalence of *Cryptosporidium* spp. was 14.5% (10/69) and was lower than that in foxes (38.5%; host species × *Cryptosporidium* INFECTION: *χ*^2^_1_ = 8.68, *p* < 0.001). Only one faecal sample from wolves was positive for *G. intestinalis* (1/69 = 1.51%; assemblage D, 96.99% identity to the sequence from dog, Brasil, GenBank Acc No. EF507620) in comparison to the 15.41% of *Giardia*-positive samples from foxes (HOST SPECIES × *Giardia* INFECTION: *χ*^2^_1_ = 8.38, *p* < 0.001).

### 3.3. Comparison of the Prevalence of Cryptosporidium/Giardia Infection Between Red Fox and Wolf

A significant difference between the prevalence of *Cryptosporidium* spp. infection between two canid species was observed. Prevalence of *Cryptosporidium* spp. was much higher in red fox (38.5%) compared to the grey wolf population (14.5%) (Host × *Cryptosporidium* spp. presence/absence: χ^2^_1_ = 8.68, *p* < 0.001. A similar trend was observed for *G. intestinalis*: the prevalence of that pathogen was higher in red foxes in comparison to the grey wolf population (15.4% vs. 1.5%, respectively; Host × *G. intestinalis* presence/absence: χ^2^_1_ = 8.38, *p* < 0.001.)

### 3.4. Phylogenetic Analysis and Molecular Identification of Cryptosporidium spp.

#### 3.4.1. Foxes

Ten products of nested PCR were successfully sequenced. *Cryptosporidium canis* was detected in four foxes: in a male from the Lower Silesia Voivodeship (100% identity to *C. canis* from a wild dog, Australia, MG516774); in one male and one female from Warmia-Masuria Voivodeship (98.4% identity to the sequence of *C. canis* derived from a dog from China, [KU608308] and 99.8% identity to the sequence of *C. canis* from a farmed Arctic fox from China, [KU215430]); and in one female from the Kuyavia-Pomerania Voivodeship (98.57% sequence identity to *C. canis* from a fox from China, [ON832696]). Two representative *C. canis* sequences were deposited to GenBank under accession numbers PV545563 and PV546873.

*Cryptosporidium baileyi* (acc no PV550074) was identified in one female from Warmia-Masuria Voivodeship (essentially identical to the *C. baileyi* from a chicken from China, HM002495, and *C. baileyi* from *Columba livia* from Iraq, KT151542).

One *Cryptosporidium* sp. sequence (PV569565) obtained from a male from Masovia Voivodeships revealed 96.1% identity to the sequence from *Microtus arvalis* from the Czech Republic (*Cryptosporidium* spp. vole genotype VII, MH145333) and 95.7% identity to the *Myodes gapperi* from the USA (KY644629).

The remaining four sequences (from females from Greater Poland, Łódź and two from Masovia Voivodeships) were classified as *Cryptosporidium* spp. because of insufficient quality of sequences to enable unambiguous identification of the *Cryptosporidium* species. However, one sequence (PV565730) derived from a female from the Masovia Voivodeship displayed 95.15% identity to *C. hominis* from a human subject in China (MT757968).

**Figure 4:**
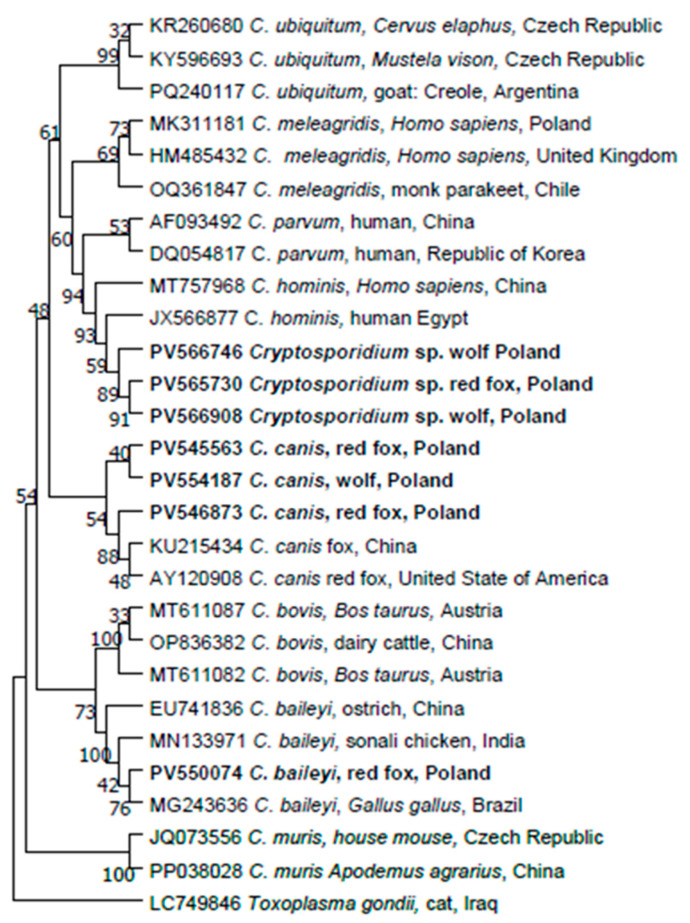
Molecular phylogenetic analysis of *18S* gene fragment of *Cryptosporidium* spp. (600 bp). The evolutionary history was inferred by using the maximum likelihood method and Kimura 2parameter model. The tree with the highest log likelihood (−2182.52) is shown. The percentage of trees in which the associated taxa clustered together is shown next to the branches. Initial tree(s) for the heuristic search were obtained automatically by applying neighbour-join and BioNJ algorithms to a matrix of pairwise distances estimated using the maximum composite likelihood (MCL) approach, and then selecting the topology with superior log likelihood value. The rate variation model allowed for some sites to be evolutionarily invariable ([+1], 18.94% sites). This analysis involved 28 nucleotide sequences. There were a total of 520 positions in the final dataset. Evolutionary analyses were conducted in MEGA11.

The phylogenetic tree obtained using the maximum likelihood method and Kimura 2-parameter model, incorporating 7 sequences obtained in this study and 21 reference sequences from GenBank, is presented in Figure 4.

#### 3.4.2. Wolves

Ten products of nested PCR were successfully sequenced. Because of insufficient quality of sequences, identification of the *Cryptosporidium* species was unsuccessful for nine samples. Only one sequence (wolf no. 7, PV554187) displayed 98.03% identity to the *C. canis* isolated from wild dogs in Australia (MG516774). However, two sequences (PV566746 and PV566908) clustered with the *C. hominis* isolated from humans from China and Egypt (Figure 4).

### 3.5. Comparison of Abundance Between Cryptosporidium Species in Red Fox Population

The highest mean abundance was observed for *C. canis* infection: 7500 ± 941.33 oocysts/1 mL of concentrated sediment. Mean abundance in the other *Cryptosporidium* positive samples detected by IFA was lower than that of *C. canis*: 1024 ± 376.53 oocysts/1 mL of concentrated sediment (main effect of *Cryptosporidium* species on abundance: *F* _5,116_ = 6.21, *p* = 0.02). MeriFluor *Cryptosporidium*/*Giardia* was unsuccessful in detecting *Cryptosporidium baileyi* and *Cryptosporidium* sp. vole genotype VII, so no mean abundance could be calculated for these species.

## 4. Discussion

In the present study, we determined the prevalence and abundance of *Cryptosporidium* and *Giardia* infections in red fox and grey wolf populations in Poland, employing three different assays. The MeriFluor IFA Kit, used in our study, is designed mainly for *C. parvum* and *C. hominis* detection and may not cross-react with other species, and therefore any failure to detect oocysts of the other *Cryptosporidium* species may be due to this limitation. Therefore, since we could not exclude the possibility that failure to detect *Cryptosporidium*/*Giardia* may have been due to the limitations of a particular test, and in order to maximise the detection of positive samples for these pathogens, we employed three methods for detecting *Cryptosporidium* and two for *Giardia*, generating for each robust values for their prevalence in foxes.

Total prevalence of *Cryptosporidium* infection in red foxes was 38.5%, and to the best of our knowledge, this is one of the highest prevalences of *Cryptosporidium* in foxes ever recorded in Europe. Prevalence of *Cryptosporidium* in foxes from Italy, Norway, Bosnia and Herzegovina and Spain did not exceed 4% [20,21,22,50]. In northwestern Spain the percentage of *Cryptosporidium*-positive faecal samples from foxes was 6.1% (out of 197 examined samples [51]). A much higher prevalence of *Cryptosporidium*, 28.3%, has been detected in foxes in Latvia [52]. In Slovakia, prevalence is almost identical to our result (38.7%) [23]. However, in another study conducted in Slovakia, *Cryptosporidium* infection was confirmed only in 2.13% of foxes [53]. Kváč et al. (2021) [53] also tested foxes from the Czech Republic and Poland for the presence of *Cryptosporidium*, but as in Norway and Italy, prevalence was recorded below 4%. In a recent study conducted in southwestern Poland (Bory Dolnośląskie area), the prevalence of *Cryptosporidium* in foxes was 12% (6 out of 50 examined animals [24]).

In this study, we examined foxes from seven voivodeships located in the east, west and south of Poland. Although we did not find any significant differences in the prevalence of *Cryptosporidium* between voivodeships, the mean abundance of *Cryptosporidium* was the highest in the Warmia-Masuria Voivodeship. The Warmia-Masuria region is an area known as the “Land of 1000 lakes”. Numerous lakes, rivers, wetlands, etc., constitute excellent environmental conditions for the survival of *Cryptosporidium*/*Giardia* (oo)cysts, facilitating the circulations/occurrence of these parasites in free-living, as well as in farm animals, living in this region [25,54,55,56].

In both foxes and wolves, *Giardia* prevalence was significantly lower than that of *Cryptosporidium*. Previous studies have revealed that *G. intestinalis* infection is generally more prevalent in young animals in comparison to adult individuals, causing watery diarrhoea, weight loss, failure to gain weight, vomiting and rapid dehydration [21,52,57]. In this study faecal samples were obtained from adult foxes and wolves, which could explain the lower *G. intestinalis* prevalence compared to that of *Cryptosporidium* sp.

Only very limited data are available for the presence of *Cryptosporidium* and *Giardia* in wolf populations. We examined 69 wolf faecal samples from the Warmia-Masuria Voivodeship and found that *Cryptosporidium* prevalence was higher than *G. intestinalis* (14.5% vs. 1.5%, respectively). Our results contrast with the result obtained for wolves from Southwestern Europe (Italy, Spain, Portugal), which followed an opposite trend: *Cryptosporidium* was detected in 3.5% of samples and *G. intestinalis* in 40.3% of wolf faecal samples [58]. Other studies conducted in Portugal and Croatia have revealed also that *G. intestinalis* is more prevalent than *Cryptosporidium* in wolf populations living in these countries [26,59].

In a previous study carried out in the Warmia-Masuria region in NE Poland, *Cryptosporidium* was detected in 35.7% of wolf faecal samples (*C. parvum* genotype 2 (bovine)) [25]. In other regions in NE Poland (Puszcza Piska and Napiwodzko-Ramuckie forests), 54.9% of wolf samples have been recorded as positive for *C. parvum* oocysts and 46.7% for *Giardia* spp. cysts [60]. Despite these differences, our results have confirmed the long-term occurrence of both intestinal protozoans in wolf populations in Poland.

In the present study we identified *C. canis*, *C. baileyi* and *Cryptosporidium* sp. vole genotype VII in red foxes in Poland. Various species of *Cryptosporidium* (*C. parvum*, *C. canis*, *C. felis*, *C. alticolis*, *Cryptosporidium* vole genotype II) have been reported previously in red foxes [22,24,61]. The diversity of *Cryptosporidium* species in foxes may result from two main factors: (1) the presence of generalist species (*C. parvum*) or specialists associated with canids (*C. canis*) and (2) detections of species associated with fox diet. Small mammals constitute the main prey of red foxes [62,63], which may explain the detection of *Cryptosporidium* species commonly occurring in rodent populations, such as *C. alticolis*, *Cryptosporidium* vole genotype II [24] and *Cryptosporidium* vole genotype VII (present study). Whether rodent-associated *Cryptosporidium* spp. effectively infect foxes and multiply in fox enterocytes, or are just passed through the intestinal tract, remains an open question. Experimental confirmation is required to verify this issue.

In our study the abundance of the canid specialist, *C. canis*, was much higher than the abundance of other species (7500 ± 941.33 vs. 1024 ± 376.53 oocysts/1 mL of concentrated sediment, respectively), which suggests that rodent-associated species/genotypes are perhaps less successful at invading and multiplying in fox enterocytes. The high abundance of *C. canis* oocysts detected by the IFA method confirmed the status of the red fox as a host for this species.

A particularly interesting finding of our study was the detection of *C. bailey* in one red fox from the Warmia-Masuria Voivodeship. *Cryptosporidium baileyi* is a bird-specific species occurring worldwide [64], including in chicken farms, and is responsible for large economic losses in poultry [65,66,67]. *Cryptosporidium baileyi* infections, causing respiratory cryptosporidiosis, have been reported in immunocompetent patients in Poland and Slovakia [68,69]. Our finding is the first report of the detection of *C. baileyi* in free-living red foxes.

*Cryptosporidium canis* occurs commonly in wild mesocarnivore populations: red foxes and wolves (present study), minks and racoon dogs [70,71] but is also detected frequently in domestic dogs [72,73,74]. Infections with this species have been reported also in humans, including children [75,76], and therefore *C. canis* is considered to be a zoonotic species.

## 5. Conclusions

The present study has confirmed the status of the red fox as a host for *Cryptosporidium*/*Giardia* species in Poland. This is the first report of the occurrence of *Cryptosporidium baileyi* in red foxes. Our results highlight the participation of wolves in the circulation of intestinal parasites of veterinary and medical significance in the natural environment, and due to the status of the wolf as a protected species, studies of the parasitic fauna of wolves are crucial for a better understanding of the wolf’s role as a reservoir for parasites.

## Figures and Tables

**Table 1:** Prevalence of *Cryptosporidium* spp. and *G. intestinalis* between foxes from examined voivodeships.

	Lower Silesia	Kuyavia-Pomerania	Łódź	Lesser Poland	Masovia	Warmia-Masuria	Greater Poland	Total
*Cryptosporidium* spp. (%)	29.41 (5/17)	23.53 (4/17)	35.71 (10/28)	12.5 (1/8)	60 (12/20)	50 (10/20)	42.86 (3/7)	38.46 (45/117)
*Giardia intestinalis*	17.56 (3/17)	5.88 (1/17)	7.14 (2/28)	0	30.00 (6/20)	30.00 (6/20)	0	15.38 (18/117)

## Data Availability

We declare all data are being provided within this manuscript.
